# Experimental investigation and quantitative prediction in interference-fit size of CFRP riveted joints under a transversal ultrasonic vibration-assisted riveting

**DOI:** 10.1038/s41598-023-41578-4

**Published:** 2023-09-02

**Authors:** Xingxing Wang, Yunyang Shi, Haicheng Pan, Yegao Chen

**Affiliations:** 1 College of Mechanical and Electrical Engineering, Suqian University, Suqian No. 399, Sucheng District, Huanghe Street, Suqian, 223800 China; 2 College of Mechanical and Electrical Engineering, Nanjing University of Aeronautics and Astronautics, Nanjing, 210016 China

**Keywords:** Aerospace engineering, Mechanical engineering, Composites, Mechanical properties, Metals and alloys

## Abstract

In this study, a transversal ultrasonic vibration-assisted riveting (TUVAR) process was developed to improve the uniformity of CFRP riveted lap joint interference-fit size, which provided a possibility for the quantization of riveted joint interference-fit sizes. The relationship between the process parameters of vibration amplitude, vibration duration, and roughness with interference-fit sizes by algorithms, through the minimum coefficient variance of interference-fit size (*I*_*CV-min*_) to confirm the riveting process parameters of the quantized average interference-fit sizes (*I*_*A*_). The experimental verification results showed that the mean absolute percentage error of measured *I*_*A*_ and predicted *I*_*A*_ is less than 10%. Furthermore, the tensile tests were carried out to investigate the effect of interference-fit size {1.4%, 1.6%, 1.8%, and 2.0%} on mechanical performances of CFRP riveted lap joints by TUVAR, and the tensile strength presents first-up then down with the interference-fit size increase, the maximum ultimate tensile strength is the riveted lap joint with the interference-fit size of 2.0%. Hence, the quantitative optimization method can well predict the riveting process parameters corresponding to the most uniform interference-fit size.

## Introduction

Carbon fiber reinforced plastics (CFRP) has been widely applied in airplane and transportation, and the potential difference in CFRP and metal limits the fastener material. Usually, titanium alloy is adopted as the fastener material, such as high lock bolts, blind rivets, and rivets. Therefore, the demand for titanium alloy rivets will be increasing due to their excellent corrosion resistance, superior intensity, and low cost. Meanwhile, the difficult deformation and sensitivity to a strain rate of titanium alloy rivets are easy to result in driven head cracks^1^ and non-uniform interference-fit size^2^, which need to be solved. How to improve the interference-fit size uniformity of CFRP laminates riveted lap joints interference-fit size connection and the quantification of riveting interference-fit size have attracted much attention. A transversal ultrasonic vibration-assisted riveting process is developed to improve the plasticity of titanium alloy rivet and investigate the effect of amplitude, vibration time, and roughness on interference-fit size because the advantages of ultrasonic vibration-assisted forming technology can not only improve the metallic plasticity^3, 4^, but also mechanical properties^5, 6^. Hu et al.^7^ found that the ultrasonic dynamic impact effect can significantly improve the plasticity of metal and reduce the surface roughness of specimens. Zhou et al.^8^ investigated the effect of ultrasonic vibration on aluminum and titanium of lightweight metals, and the results showed that ultrasonic softening is affected by not only vibration amplitude but also frequency, and ultrasonic vibration also can refine the grains for lightweight metals. Djavanroodi et al.^9^ developed the superimposing ultrasonic vibration-assisted equal channel angular pressing, which can not only decline the punch load but also improve the mechanical properties. Zhuang et al.^10^ researched the mechanism of ultrasonic-assisted compression and found that both the flow stress and surface roughness declined.

Although ultrasonic vibration technology has been applied for decades, seldom scholars applied it in the riveting process. Interference-fit size is an important evaluation standard for the performance of riveted joints, hence it is necessary to investigate the effect of ultrasonic vibration parameters on interference-fit sizes. Lots of scholars have researched the relationship between interference-fit size and mechanical performance. Jiang et al.^11^ comprehensively studied the effect of different sidewall intersection angles of rivet dies on interference-fit size, and the fatigue tests showed that the pull-out fatigue property of the 80° driven head was the best due to its more uniform and moderate interference-fit size. Wei et al.^12^ carried out the fatigue tests for the CFRP joints with blind bolts of different interference-fit sizes, and found that the best interference-fit size of CFRP bolted lap joints under different cyclic stress were different, such as, the bearing stress was more than 660 MPa, the best corresponding interference-fit size was 1.8%. Li et al.^13^ studied the effect of interference-fit size and different percentages of ultimate bearing strength on the bearing fatigue response of CFRP/Ti alloy bolted joints and found that the fatigue life of interference-fit size under high tensile stress was 2.1%, the longest fatigue life of interference-fit size under low cycle stress was 1.2%. Hu et al.^14^ investigated the relationship between interference-fit size and stiffness of CFRP bolted lap joints, the results showed that the interference-fit size corresponding to the highest stiffness of CFRP bolted lap joints was 1.2%. To sum up, the effect of the interference-fit size of lap joints with the aluminum alloy rivet, high-locking bolt, and blind bolt et al. on mechanical properties are different, but the interference-fit sizes of CFRP riveted lap joints with better mechanical performances are lower than 2%.

An inhomogeneous expansion of the rivet shaft is the primary cause to restrict the improvement of riveting quality, which has attracted much attention. The non-uniform deformation of titanium alloy rivets is much worse than aluminum due to its poor plasticity. However, the unclear effects of ultrasonic vibration energy on interference-fit size distribution, hence it is necessary to explore the relationship between the ultrasonic vibration parameters and the interference-fit size and improve the uniformity of the interference-fit size. Scholars have explored ways to improve the uniform interference-fit size in both process and algorithm optimization. Skorupa et al.^15^ designed a novel rivet with a compensator, and a better uniform interference-fit size and fatigue performance were achieved. Cui et al.^16^ investigated the interference-fit size and forming mechanism of adiabatic shear bands with trapezoidal section rivet dies, and found that a reasonable riveting dies angle can effectively improve the uniformity of interference-fit size. Wang et al.^17^ proposed an integration method to optimize riveting parameters of pressure, riveting time, and dwelling time using the finite element method and Kriging metamodels with particle swarm optimization, and the optimized riveting parameters could improve the deformation homogeneity. Cao et al.^18^ developed a special gasket to restrain the expansion of the rivet shaft and inhibited the hole damage of CFRP caused by a large interference-fit size. In addition, scholars^19, 20^ also proposed the electromagnetic riveting process to achieve the uniform expansion of the aluminum alloy rivet shaft. Although the uniformity of interference-fit size in the riveted lap joints can be well improved, the quantitative and control method of interference-fit size is still lacking.

In this study, the unclear effects of ultrasonic parameters on the interference-fit size and mechanical performances of CFRP riveted lap joints were explored. First, the interference-fit sizes of CFRP riveted lap joints are measured, and introduced the coefficient of variance and the average value of interference-fit size. Second, the response surface algorithm, interpolation algorithm, and random forest are adopted to quantify and control the interference-fit size of CFRP riveted lap joints, and the optimized interference-fit sizes {*I*_*A*_, *I*_*CV-min*_, (*C*_*1*_*, C*_*2*_, *C*_*3*_)} are verified by experiments. Furthermore, the tensile tests were carried out, and the fracture mode and morphology were analyzed and observed.

## Experimental procedures

### Transversal ultrasonic vibration-assisted riveting system

**Figure 1:**
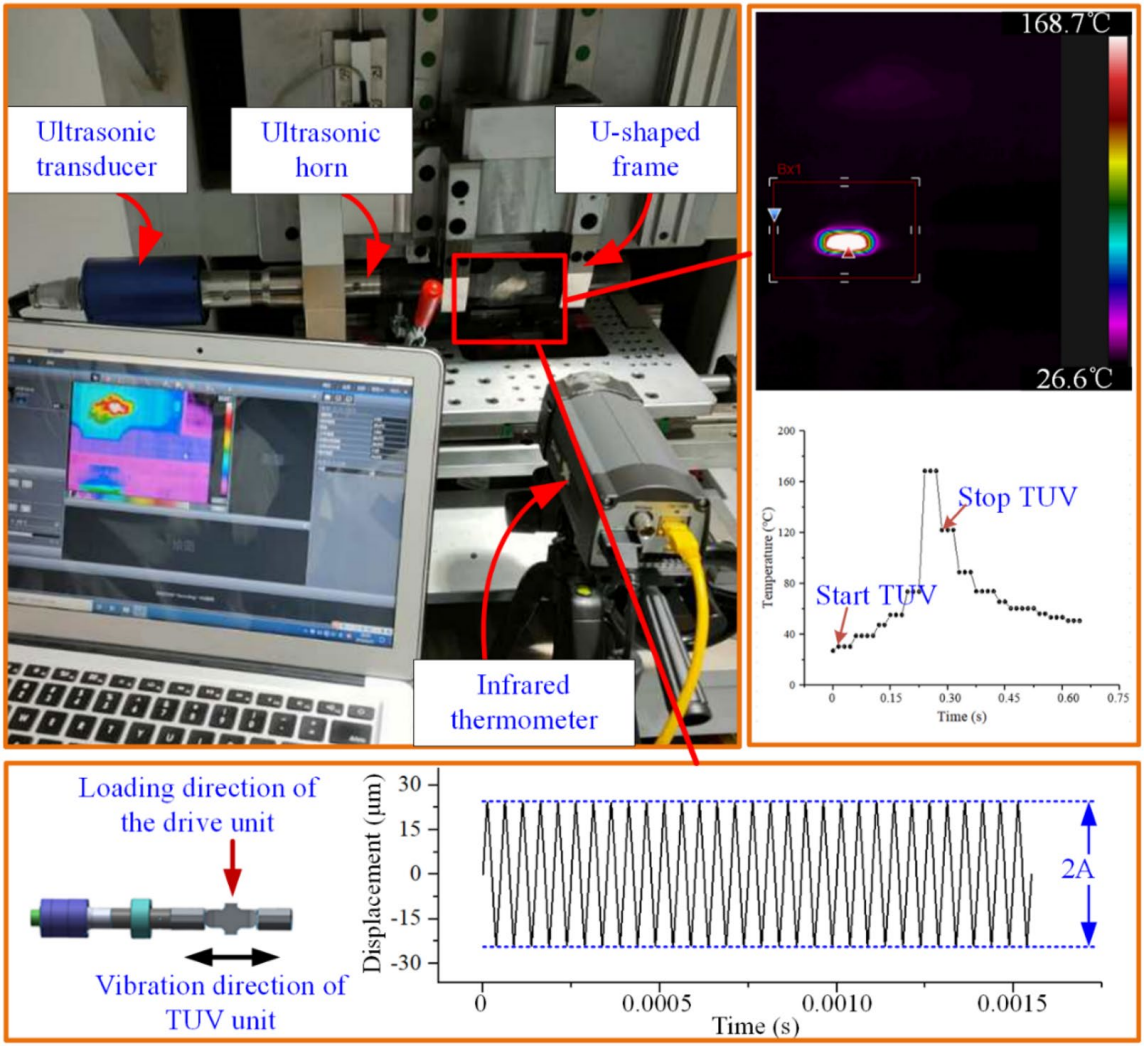
The schematic of the TUVAR system^27^.

**Figure 2:**
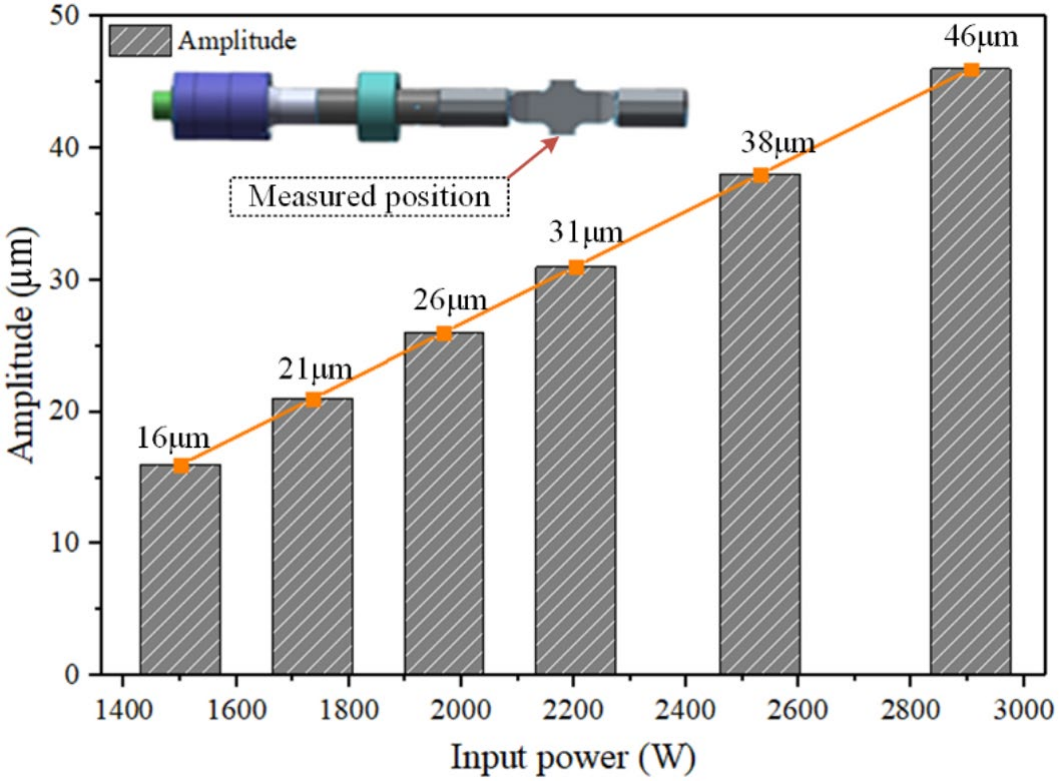
The relationship between input power and amplitude^27^.

In Fig. 1, the developed transversal ultrasonic vibration-assisted riveting (TUVAR) system mainly consists of a drive unit, an ultrasonic vibration assistance unit, and a data acquisition unit. The ultrasonic vibration assistance unit is in charge of providing acoustic energy. Specifically, when connecting with the power, the ultrasonic generator transfers the low-frequency electric energy to high-frequency electric energy. Then the ultrasonic transducer is used to transfer the electric energy to mechanical energy, furtherly the ultrasonic horn amplifies the vibration amplitude. Finally, the high-frequency mechanical vibration with an amplified amplitude is applied to rivets, until the compression is completed. In the TUVAR process, the vibration amplitude has a significant effect on the plasticity improvement of the Ti-alloy rivet based on the previous study^4^, which would further affect the rivet deformation and joint performance. The amplitudes with different power were measured by a dial indicator (Syntek-JR3), and the measured results are shown in Fig. 2.

### Specimen preparation

**Figure 3:**
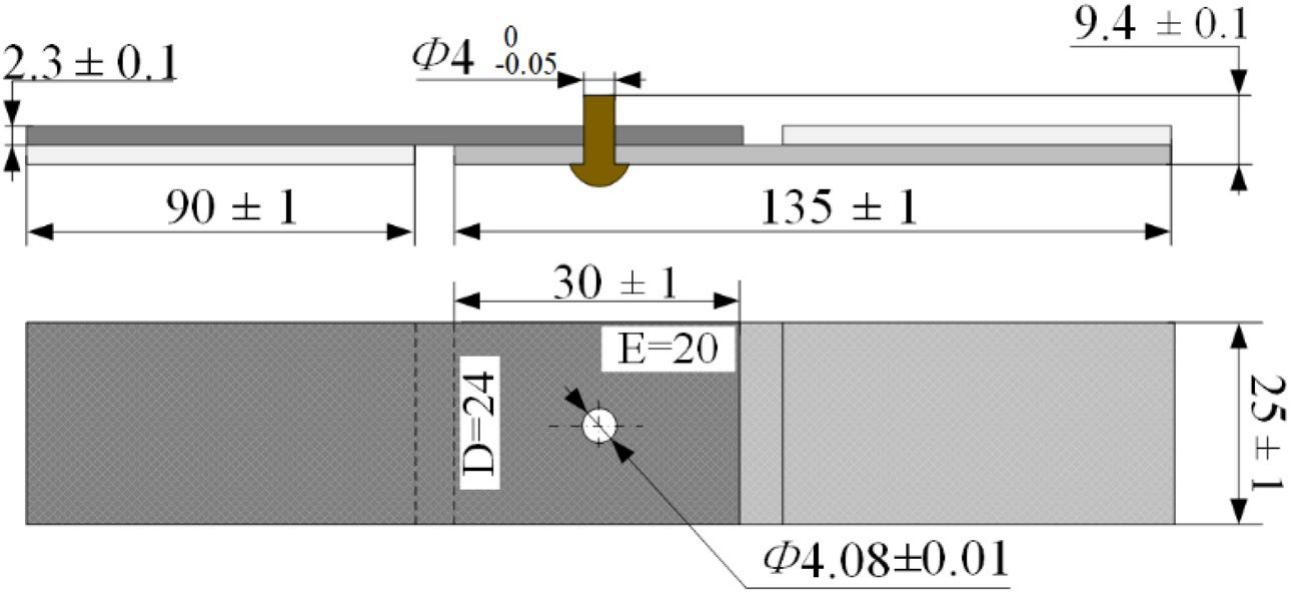
Dimensions of the CFRP laminates riveted lap joint (dimension in mm).

The T700 CFRP laminates and Ti-45Nb rivets were selected for interference-fit sizes optimization and tensile specimens. T700 CFRP was using a unidirectional carbon fiber/epoxy with a thickness of 0.15 mm per ply (provided by GW COMPOS Company Ltd., China). The fabricated T700 CFRP laminate processes a thickness of 2.3 mm with 16 piles, the ply orientation of T700 CFRP is[0°/90°/45°/− 45°/− 45°/45°/90°0°]_2 s_, and the weight fraction of carbon fiber is about 60%. Material properties of the fabricated CFRP laminates are presented in Table 1. Moreover, the Fabricated Ti-45Nb rivets (provided by CAG Company Ltd., China) were annealed by heating in a vacuum (less than 0.1um mercury) to a temperature within the range of 1450–1600 °F, holding at heat for sufficient time to produce a recrystallized structure that will meet the requirements of 3.5. Material properties of the Ti-45Nb are presented in Table 1. In addition, the sizes of CFRP riveted specimens according to the ASTM D5661^21^ are shown in Fig. 3, and *W*/*D* ≥ 6, *E*/*D* ≥ 3.

**Table 1:** Mechanical properties of the sample^4^.

CFRP laminates		Ti-45Nb	
Property	Value	Property	Value
Resin content (%)	40	Density [g/cm3]	5.7
Tensile strength (Mpa)	2300	Poisson ratio	0.34
Tensile modulus (Gpa)	115	Tensile modulus [GPa]	62
Flexural strength (Mpa)	1250	Yield strength [MPa]	425
Compressive strength (Mpa)	1050	Tensile strength [MPa]	570
Interlaminar shear strength (Mpa)	55

### Experimental procedure

**Figure 4:**
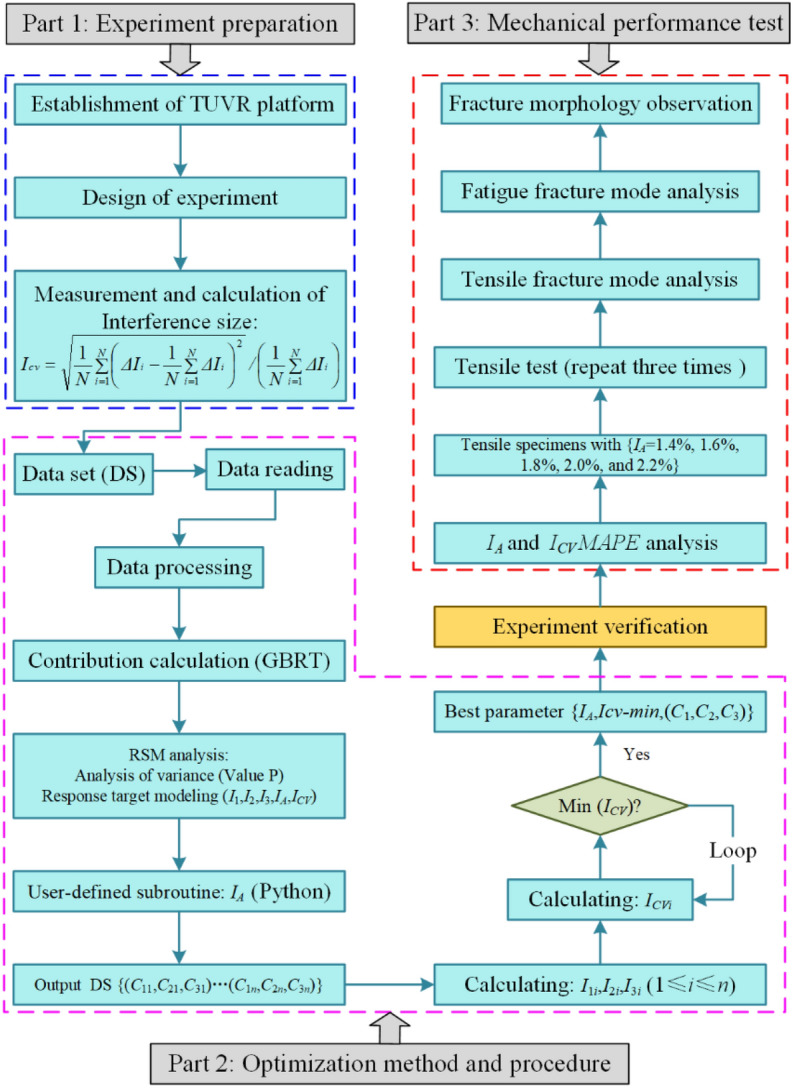
Experimental process of interference-fit size optimization.

**Figure 5:**
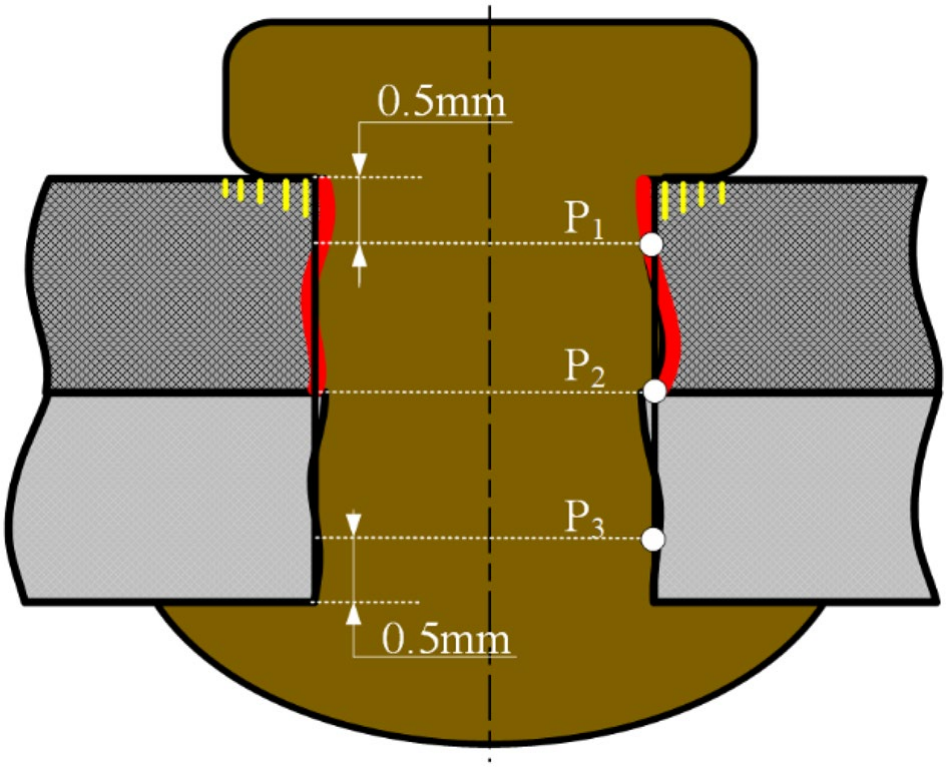
Experimental process of interference-fit size optimization^24^.

The experimental procedure is divided into three parts, as shown in Fig. 4. Part 1: the experiments of orthogonal and single factor were carried out, and the diameters of the deformed rivet bar were measured with three positions^1, 18^, as shown in Fig. 5. Furthermore, the relative interference-fit size {*I*_1_, *I*_2_, *I*_3_} of three positions were calculated by Eq. (1), and the average relative interference size (*I*_*A*_) and coefficient of variance of relative interference size (*I*_*CV*_) were introduced, the corresponding calculation equations are following in Eqs. (2, 3), respectively. Part 2: the {*I*_1_, *I*_2_, *I*_3_, *I*_*A*_, *I*_*CV*_} data set was analyzed by surface response algorithm (RSA), the relationship between ultrasonic process parameters vibration amplitude (*C*_1_), vibration time (*C*_2_), and roughness of rivet end surface (*C*_3_) with {*I*_1_, *I*_2_, *I*_3_, *I*_*A*_, *I*_*CV*_} are established, and the effective weight and *P* value were analyzed. Moreover, the random forest algorithm and extremely randomized trees are combined to evaluate the contribution value of process parameters, the corresponding calculation equations as follows from Eqs. (4, 5, 6, 7, 8). Furthermore, the minimum coefficient of variance of relative interference-fit size (*I*_*CV-min*_) and *I*_*A*_ was as response targets, the user-defined subroutine using Python to optimize the (*C*_1_, *C*_2_, *C*_3_) by interpolation algorithm. The optimized {*I*_*A*_, *I*_*CV-min*_, (*C*_1_, *C*_2_, *C*_3_)} groups were verified by experiment, and the mean absolute percentage error (*MAPE*) of experimental and predicted {*I*_*A*_, *I*_*CV-min*_} was calculated. Part 3: the tensile tests specimens with {*I*_*A*_ = 1.4%, 1.6%, 1.8%, 2.0%, and 2.2%} were carried out, the results of tensile ultimate strength were analyzed, and the fracture morphology was observed by HIROX-2000 ultra-depth three-dimensional microscope.

1 $$I = \frac{{D_{1} - D_{0} }}{{D_{0} }} \times 100\%$$

where *D* and *D*_0_ are the diameters of the deformed rivet shaft and initial riveting hole.

2 $$I_{A} = \frac{1}{N}\sum\limits_{i = 1}^{N} {I_{i} }$$

where *I*_*A*_ presents the average relative interference size; *N* is the number of the measured positions of each deformed rivet; *I*_*i*_ is the relative interference size with different measured positions.

3 $$I_{CV} = \sqrt {\frac{1}{N}\sum\limits_{i = 1}^{N} {\left( {\Delta I_{i} - \frac{1}{N}\sum\limits_{i = 1}^{N} {\Delta I_{i} } } \right)^{2} } } /\left( {\frac{1}{N}\sum\limits_{i = 1}^{N} {\Delta I_{i} } } \right)$$

where *I*_*CV*_ presents the coefficient of variance of relative interference size, i.e. the evaluation index of interference-fit size uniformity. To accurately evaluate the contribution value of the TUVAR process parameters, the random forest, and extremely randomized trees are adopted to quantize the weight magnitude of the process parameters^22, 23^. Considering the disadvantages of the algorithm analysis, the random forest and extremely randomized trees are used to authenticate and take the mean of multiple cycles superposition calculation.

## Experimental results and discussions

### Interference-fit size analysis

Interference-fit size has a significant effect on the mechanical properties of CFRP riveted lap joints^25^. The relative interference-fit sizes {*I*_1_, *I*_2_, *I*_3_} correspond to the measured positions (P_1_, P_2_, P_3_) based on Fig. 5. Each position of the deformed rivet bar was repeated three times at intervals of 120° using a Vernier caliper with the precision of 0.01 mm. Furthermore, the {*I*_1_, *I*_2_, *I*_3_, *I*_*A*_, *I*_*CV*_} are calculated with Eqs. (1, 2, 3), respectively. The calculated results of {*I*_1_, *I*_2_, *I*_3_, *I*_*A*_, *I*_*CV*_} were listed in Table 2, where scheme 0 is the conventional riveting process without ultrasonic vibration, the scheme 1–28 are the transversal ultrasonic vibration-assisted riveting (TUVAR). But scheme 16 is disqualification, because of the severe damage around the CFRP riveted hole. It could be seen that the *I*_*CV*_ of scheme 0 is 0.369, which is much larger than the *I*_*CV*_ of TUVAR schemes. Therefore, the uniformity of the interference-fit size can be well improved by transversal ultrasonic vibration. It also could be seen that the *I*_*A*_ of scheme 16 is 0.0504, which is much larger than the upper limit value of 0.02 of interference-fit size for CFRP laminates riveted lap joints. It is because the acoustic energy with the largest amplitude 31 μm and vibration time 2.0 s, which make the Ti-45Nb rivet soften and promote the severe deformation of the rivet, and leadqa to the *I*_*A*_ of scheme 16 remarkable increase.

**Table 2:** The calculated results of the {*I*_1_, *I*_2_, *I*_3_, *I*_*A*_, *I*_*CV*_}.

Scheme	Amplitude (um)	Time(s)	Roughness (um)	*I*_*1*_	*I*_*2*_	*I*_*3*_	*I*_*A*_	*I*_*CV*_	Quality
0	0	1.2	2.0	0.0202	0.0074	0.0154	0.0143	0.3690	√
1	16	0.8	1.9	0.0147	0.0123	0.0147	0.0139	0.0832	√
2	16	1.2	2.2	0.0160	0.0123	0.0172	0.0152	0.1378	√
3	16	1.6	2.7	0.0172	0.0160	0.0184	0.0172	0.0583	√
4	16	2.0	3.6	0.0184	0.0172	0.0209	0.0188	0.0813	√
5	21	0.8	2.2	0.0172	0.0160	0.0184	0.0172	0.0583	√
6	21	1.2	1.9	0.0160	0.0147	0.0184	0.0164	0.0935	√
7	21	1.6	3.6	0.0233	0.0197	0.0209	0.0213	0.0720	√
8	21	2.0	2.7	0.0184	0.0172	0.0197	0.0184	0.0544	√
9	26	0.8	2.7	0.0172	0.0160	0.0160	0.0164	0.0354	√
10	26	1.2	3.6	0.0221	0.0209	0.0197	0.0209	0.0480	√
11	26	1.6	1.9	0.0233	0.0221	0.0209	0.0221	0.0454	√
12	26	2.0	2.2	0.0172	0.0147	0.0197	0.0172	0.1166	√
13	31	0.8	3.6	0.0160	0.0147	0.0160	0.0156	0.0372	√
14	31	1.2	2.7	0.0233	0.0209	0.0209	0.0217	0.0534	√
15	31	1.6	2.2	0.0307	0.0295	0.0184	0.0262	0.2108	√
16	31	2.0	1.9	0.0602	0.0565	0.0344	0.0504	0.2262	╳
17	16	1.2	2.0	0.0176	0.0143	0.0160	0.0160	0.0844	√
18	21	1.2	2.0	0.0184	0.0168	0.016	0.0171	0.0585	√
19	26	1.2	2.0	0.0209	0.0143	0.0168	0.0173	0.1570	√
20	31	1.2	2.0	0.0274	0.0266	0.0249	0.0263	0.0396	√
21	16	0.8	2.0	0.0151	0.011	0.0135	0.0132	0.1278	√
22	16	1.2	2.0	0.0176	0.0143	0.016	0.0160	0.0844	√
23	16	1.6	2.0	0.0192	0.016	0.0200	0.0184	0.0939	√
24	16	2	2.0	0.0217	0.0175	0.0184	0.0192	0.0940	√
25	16	1.2	1.9	0.0172	0.0147	0.0164	0.0161	0.0647	√
26	16	1.2	2.2	0.0172	0.0130	0.0140	0.0147	0.1216	√
27	16	1.2	2.7	0.0174	0.0147	0.0164	0.0162	0.0689	√
28	16	1.2	3.6	0.0172	0.0140	0.0164	0.0159	0.0857	√

**Figure 6:**
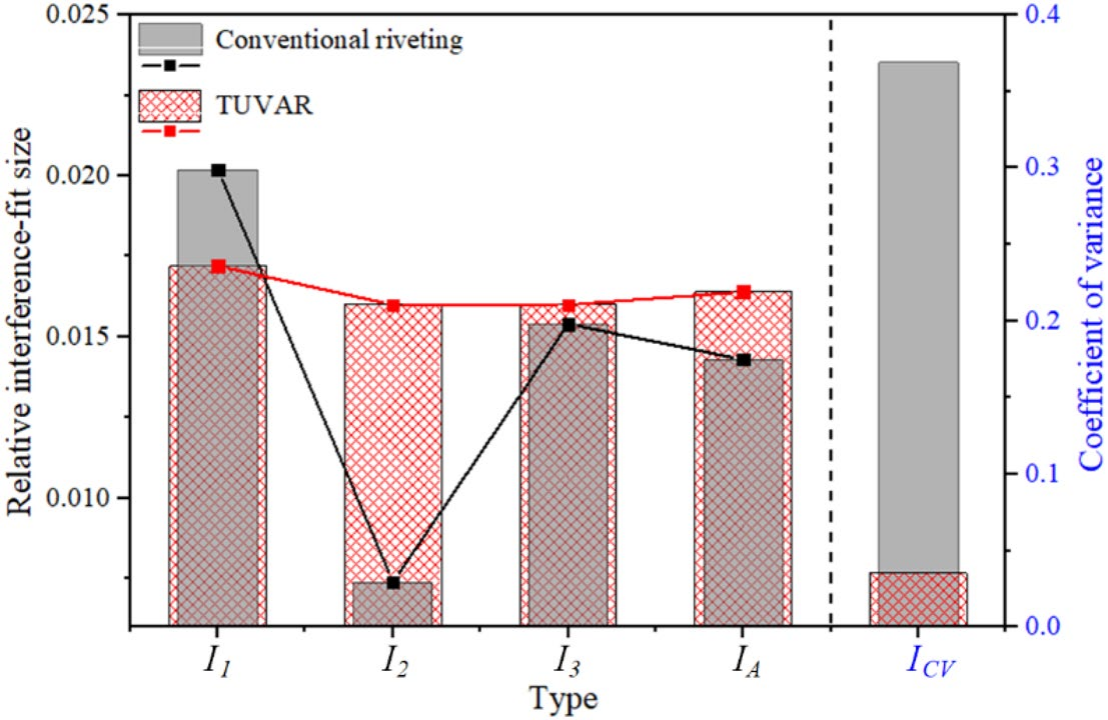
The comparison of {*I*_1_, *I*_2_, *I*_3_, *I*_*A*_, *I*_*CV*_}: conventional riveting (scheme 0), TUVAR (scheme 9).

As shown in Fig. 6, the {*I*_1_, *I*_2_, *I*_3_, *I*_*A*_, *I*_*CV*_} of conventional riveting (scheme 0) and the typical TUVAR (scheme 9) at the same pressure and velocity are compared. It could be seen that the interference-fit size of P_1_ declined, and the interference-fit size of P_2_ improved under TUVAR. Hence, the TUVAR can well overcome an aperture constraint and promote the hole entrance material to flow into the P_2_ region, which improves the uniformity of interference-fit size a lot, and it has been identified from the microscale^24^. Besides, the *I*_*A*_ of TUVAR increases by about 14.7%, because more material from the driven head flows into the riveted hole under TUVAR. The comparison of *I*_*CV*_ of conventional riveting and TUVAR showed that the *I*_*CV*_ decreases about 9 times. Hence, a much more uniform interference-fit size of CFRP riveted lap joint can be achieved with the assistance of transversal ultrasonic vibration, which contributes a possibility for the interference-fit size quantization of riveted lap joint.

### Interference-fit size optimization by response surface algorithm

The user-defined RSA is used for the process data set in Table 2, and the variance analysis result (Model, *C*_1_, *C*_2_, *C*_3_) of the {*I*_1_, *I*_2_, *I*_3_, *I*_*A*_, *I*_*CV*_} are displayed in Table 3. It is acknowledged that the *P* ≤ 0.05, the model, and factors are significant. The predicted models for {*I*_1_, *I*_2_, *I*_3_, *I*_*A*_, *I*_*CV*_} are shown in Eqs. (4, 5, 6, 7, 8). According to the evaluation index of *P*-value, the models of {*I*_1_, *I*_2_, *I*_3_, *I*_*A*_} are significant, except the *I*_*CV*_. It means that the model relationship between the *I*_*CV*_ and (*C*_1_, *C*_2_, *C*_3_) is poorly reliable, hence the process parameter (*C*_1_, *C*_2_, *C*_3_) cannot be directly optimized by the *I*_*CV*_ model. Therefore, the predicted function of Eq. (8) cannot be straightly applied to the reserve solving and optimizing the TUVAR process parameters, Eq. (3) is still adopted to calculate the *I*_*CV*_.

**Table 3:** Variance analysis of the {*I*_1_, *I*_2_, *I*_3_, *I*_*A*_, *I*_*CV*_}.

Type	Factor	Freedom	Seq SS	Adj SS	Adj MS	F	*P*	Significant
*I*_1_	Model	9	0.000274	0.000274	0.000030	6.02	0.001	Yes
	*C*_1_	1	0.000142	0.000075	0.000075	14.91	0.001	
	*C*_2_	1	0.000043	0.000025	0.000025	5.04	0.038	
	*C*_3_	1	0.000011	0.000009	0.000009	1.77	0.200	
*I*_2_	Model	9	0.000332	0.000332	0.000037	4.94	0.002	Yes
	*C*_1_,	1	0.000209	0.000109	0.000109	14.61	0.001	
	*C*_2_	1	0.000051	0.000033	0.000033	4.38	0.052	
	*C*_3_	1	0.000002	0.000001	0.000001	0.18	0.674	
*I*_3_	Model	9	0.000121	0.000121	0.000013	4.29	0.005	Yes
	*C*_1_	1	0.000044	0.000013	0.000013	4.04	0.060	
	*C*_2_	1	0.000052	0.000017	0.000017	5.55	0.031	
	*C*_3_	1	0.000000	0.000001	0.000001	0.24	0.628	
*I*_*A*_	Model	9	0.000218	0.000218	0.000024	7.14	0.00001	Yes
	*C*_1_	1	0.000121	0.000057	0.000057	16.75	0.001	
	*C*_2_	1	0.000048	0.000024	0.000024	7.91	0.016	
	*C*_3_	1	0.000002	0.000003	0.000003	0.83	0.375	
*I*_*CV*_	Model	9	0.016178	0.016178	0.001798	1.16	0.378	No
	*C*_1_	1	0.000047	0.001062	0.001062	0.69	0.419	
	*C*_2_	1	0.000902	0.003144	0.003144	2.03	0.173	
	*C*_3_	1	0.006364	0.001814	0.001814	1.17	0.294	

**Figure 7:**
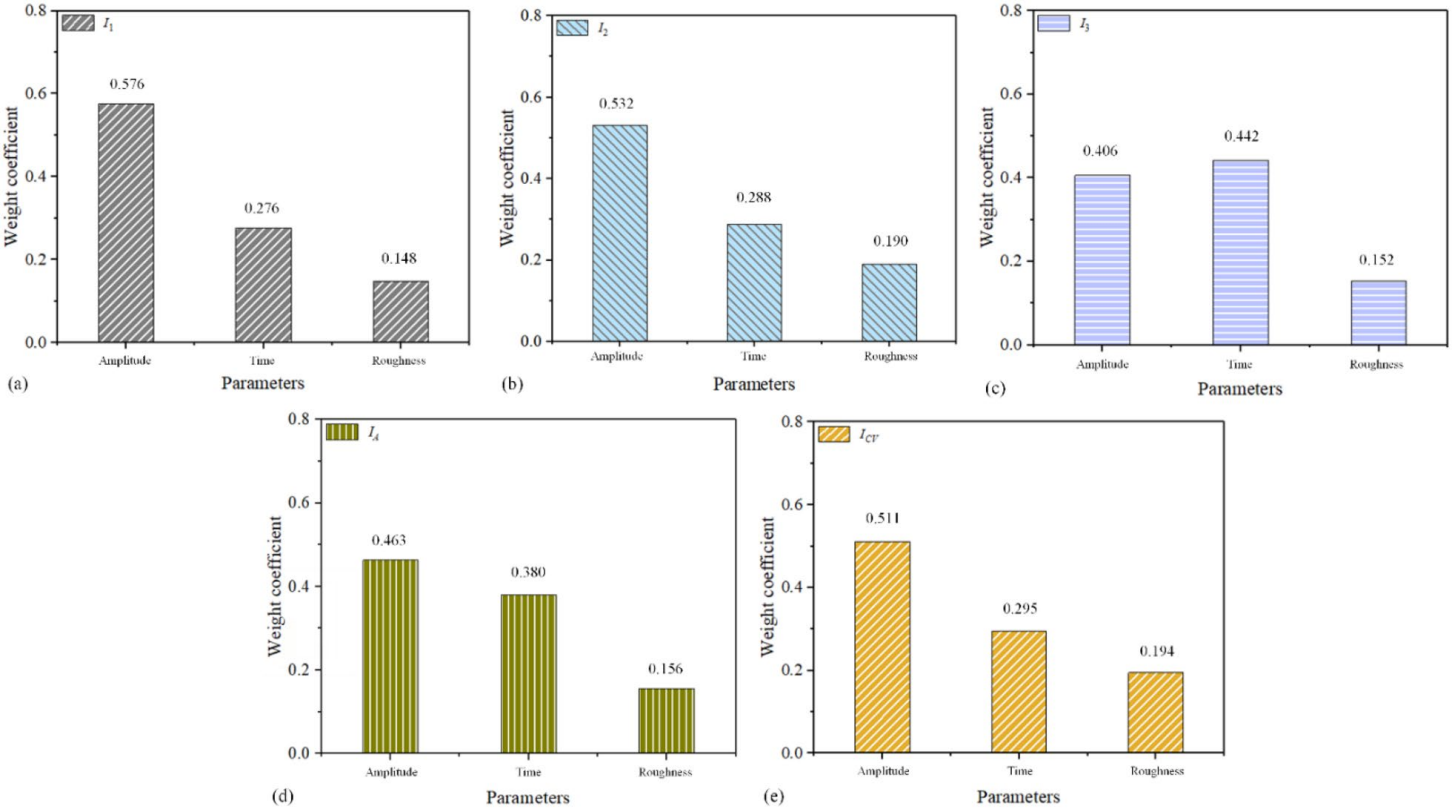
Quantitative weight coefficient: the effect weight of (*C*_1_, *C*_2_, *C*_3_) on {*I*_1_, *I*_2_, *I*_3_, *I*_*A*_, *I*_*CV*_}.

According to the independent *P*-value of (*C*_1_, *C*_2_, *C*_3_) on {*I*_1_, *I*_2_, *I*_3_, *I*_*A*_}, it could be obtained that *C*_1_ and *C*_2_ have a significant effect on {*I*_1_, *I*_2_, *I*_3_, *I*_*A*_}, except *C*_3_. For *I*_*CV*_, the independent *P*-values of (*C*_1_, *C*_2_, *C*_3_) are larger than 0.05, which is insignificant. Besides, the significance ranking of (*C*_1_, *C*_2_, *C*_3_) on {*I*_1_, *I*_2_, *I*_*A*_} is *C*_1_ > *C*_2_ > *C*_3_, and the significance ranking of (*C*_1_, *C*_2_, *C*_3_) on *I*_3_ is *C*_2_ > *C*_1_ > *C*_3_. The reason for the significance ranking of *I*_3_ is considering that the ultrasonic vibration attenuation transfer mode leads to softening effect reduction in the P_3_ area. Meanwhile, the random forest algorithm and the extremely randomized trees were used to verify and quantify the contribution values of process parameters (*C*_1_, *C*_2_, *C*_3_) for the RSA results. After several times training by random forest algorithm, the results are shown in Fig. 7. It could be seen that the contributions of process parameters (*C*_1_, *C*_2_, *C*_3_) have a good consistency with the *P* values of (*C*_1_, *C*_2_, *C*_3_) by RSA, which further identify the analysis results RSA is reliability. In Fig. 7, the effect weight values of process parameters (*C*_1_, *C*_2_, *C*_3_) for {*I*_1_, *I*_2_, *I*_3_, *I*_*A*_, *I*_*CV*_} are {*I*_1_: (0.576,0.276,0.148), *I*_2_: (0.532,0.288,0.190), *I*_3_: (0.406,0.422,0.152), *I*_*A*_: (0.463,0.380,0.156), *I*_*CV*_: (0.511,0.295,0.149)}, respectively.

**Figure 8:**
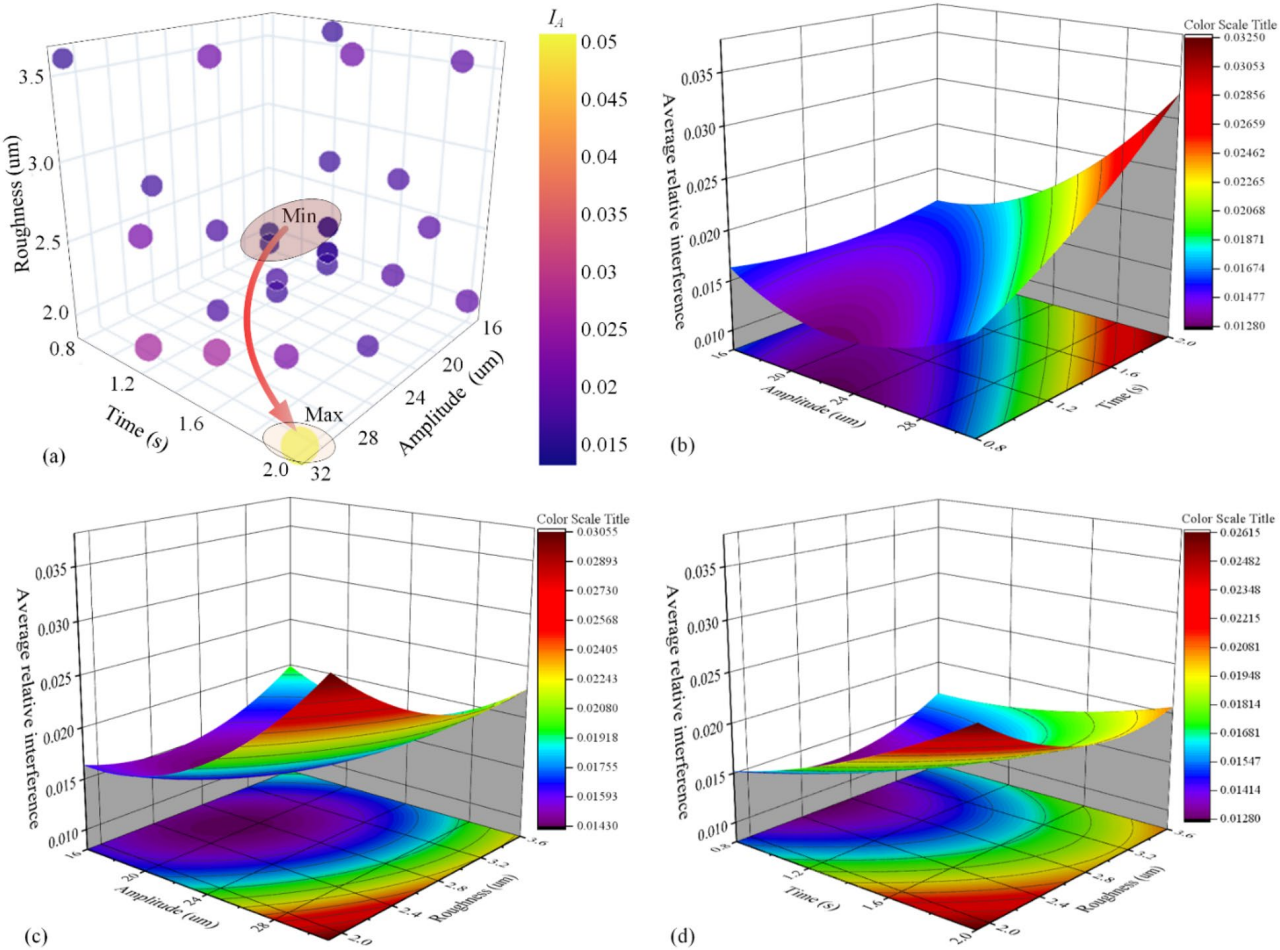
Bubble chart and RSA of *I*_*A*_: (**a**) bubble chart, (**b**) amplitude-time, (**c**)amplitude-roughness, (**d**) time-roughness.

According to the RSM analyzed results, the relationship between parameters and {*I*_1_, *I*_2_, *I*_3_, *I*_*A*_, *I*_*CV*_} are built by the least square method (as shown in Eq. 4). To reveal the interaction of process parameters (*C*_1_, *C*_2_, *C*_3_) on the *I*_*A*_, the four-dimensional bubble charts and RSA charts are combined based on experimental results and Eqs. (5, 6, 7, 8), as displayed in Fig. 8. In Fig. 8a, it could be seen that the larger of amplitude and time is, the larger *I*_*A*_ is achieved. In Fig. 8b–d, it can be seen that amplitude and time have a larger effect on *I*_*A*_, and the effect of roughness on *I*_*A*_ is minimal. Due to the insignificant effect of parameters on *I*_*CV*_, the corresponding four-dimensional bubble charts, and RSA charts are not analyzed.

4 $$y^{\prime}\left( x \right) = \beta_{0} + \sum\limits_{i = 1}^{n} {\beta_{i} x_{i} } + \sum\limits_{i = 1}^{n} {\beta_{ii} x_{i}^{2} } + \sum\limits_{i = 1}^{n} {\beta_{ij} x_{i} } x_{j} + \varepsilon$$

where *x*_*i*_ is the process parameter, *ε* is a residual error, and *β*_0_ *β*_*i*_* β*_*ii*_* β*_*ij*_ are undetermined coefficients.

5 $$\begin{aligned} I_{1} = & 0.0781838 - 0.00406381C_{1} - 0.0171465C_{2} - 0.00953967C_{3} + 0.968158 \times 10^{ - 4} C_{1}^{2} + 0.00400439C_{2}^{2} \\ & \quad + 0.00505722C_{3}^{2} + 0.00118399C_{1} * C_{2} - 0.536399 \times 10^{ - 3} C_{1} * C_{3} - 0.00540458C_{2} * C_{3} \\ \end{aligned}$$

6 $$\begin{aligned} I_{2} = & 0.0693615 - 0.00366412C_{1} - 0.0186017C_{2} - 0.00846697C_{3} + 8.84606 \times 10^{ - 5} C_{1}^{2} + 0.00362884C_{2}^{2} \\ & \quad + 0.00447198C_{3}^{2} + 0.00115261C_{1} * C_{2} - 4.9163 \times 10^{ - 4} C_{1} * C_{3} - 0.00408892C_{2} * C_{3} \\ \end{aligned}$$

7 $$\begin{aligned} I_{3} = & 0.0293963 - 3.29405 \times 10^{{{ - }4}} C_{1} - 0.00442068C_{2} - 0.00809061C_{3} + 2.56606 \times 10^{ - 5} C_{1}^{2} + 0.0021805C_{2}^{2} \\ & \quad + 0.00270885C_{3}^{2} + 0.000159745C_{1} * C_{2} - 2.98161 \times 10^{ - 4} C_{1} * C_{3} - 3.41037 \times 10^{ - 4} C_{2} * C_{3} \\ \end{aligned}$$

8 $$\begin{aligned} I_{A} = & 0.0589805 - 0.00268578C_{1} - 0.0133896C_{2} - 0.00869902C_{3} + 7.0312 \times 10^{ - 5} C_{1}^{2} + 0.00327124C_{2}^{2} \\ & \quad + 0.00407935C_{3}^{2} + 0.000832113C_{1} * C_{2} - 4.42085 \times 10^{ - 4} C_{1} * C_{3} - 0.00327818C_{2} * C_{3} \\ \end{aligned}$$

9 $$\begin{aligned} I_{CV} = & 0.654294 - 0.0338104C_{1} - 0.244662C_{2} - 0.0434314C_{3} + 5.32342 \times 10^{ - 4} C_{1}^{2} + 0.032368C_{2}^{2} \\ & \quad + 0.0136514C_{3}^{2} + 0.0100315C_{1} * C_{2} - 0.0012415C_{1} * C_{3} - 0.0142186C_{2} * C_{3} \\ \end{aligned}$$

Therefore, according to the RSA and interpolation method, a user subroutine is defined by Python. First, the *I*_*A*_ is the quantized target of interference-fit size to obtain the data set {(*C*_11_, *C*_22_, *C*_33_) … (*C*_1i_, *C*_2i_, *C*_3i_)}. Then the data set {(*C*_11_, *C*_22_, *C*_33_) … (*C*_1i_, *C*_2i_, *C*_3i_)} are transferred to Eqs. (4, 5, 6) to achieve data set {(*I*_11_, *I*_22_, *I*_33_) … (*I*_1i_, *I*_2i_, *I*_3i_)}. Furthermore, the data set {(*I*_11_, *I*_22_, *I*_33_) … (*I*_1i_, *I*_2i_, *I*_3i_)} are inputted in Eq. (3) to calculate and output data set {*I*_*CV1*_ … *I*_*CVi*_}, then looping comparison the *I*_*CV1*_ to *I*_*CVi*_ and confirming the *I*_*CV-min*_, as shown in Fig. 4. Finally, based on *I*_*CV-min*_ to confirm the optimal process parameters (*C*_1_, *C*_2_, *C*_3_). The optimized and predicted {*I*_*A-p*_, *I*_*CV-min*_, (*I*_1_, *I*_2_, *I*_3_), (*C*_1_, *C*_2_, *C*_3_)} are listed in Table 4. It could be seen that the value of *I*_*CV-min*_ is less than 0.1, except for predicted schemes 1 and 11. But the maximum deviation of (*I*_1_, *I*_2_, *I*_3_) for predicted schemes 1 and 11 are 0.0033 and 0.0048, respectively, compared with the maximum deviation of (*I*_1_, *I*_2_, *I*_3_) for conventional riveting is 0.0128, the uniformity of interference-fit size significantly improves.

**Table 4:** Predicted results of relative interference sizes by interpolation algorithm.

Predicted scheme	*I*_*A-p*_	*I*_*CV-min*_	(*I*_1_, *I*_2_, *I*_3_)	(*C*_1_, *C*_2_, *C*_3_)
1	0.013	0.1270	(0.0133, 0.0113, 0.0146)	(21.10, 0.812, 2.709)
2	0.014	0.0844	(0.0148, 0.0123, 0.0148)	(19.75, 0.800, 2.898)
3	0.015	0.0749	(0.0153, 0.0133, 0.0159)	(26.80, 0.968, 3.195)
4	0.016	0.0602	(0.0173, 0.015, 0.0157)	(27.85, 1.052, 3.141)
5	0.017	0.0294	(0.0175, 0.0163, 0.0171)	(28.15, 1.136, 3.249)
6	0.018	0.0507	(0.0189, 0.0167, 0.0181)	(26.80, 1.412, 3.330)
7	0.019	0.0659	(0.0199, 0.0172, 0.0198)	(25.90, 1.628, 3.384)
8	0.020	0.0820	(0.0219, 0.0179, 0.0202)	(25.30, 1.796, 3.465)
9	0.021	0.0876	(0.0222, 0.0182, 0.0219)	(25.00, 1.904, 3.573)
10	0.022	0.0916	(0.0238, 0.0191, 0.0230)	(25.45, 1.976, 3.573)
11	0.023	0.1001	(0.0247, 0.0197, 0.0245)	(26.35, 1.988, 3.573)

**Figure 9:**
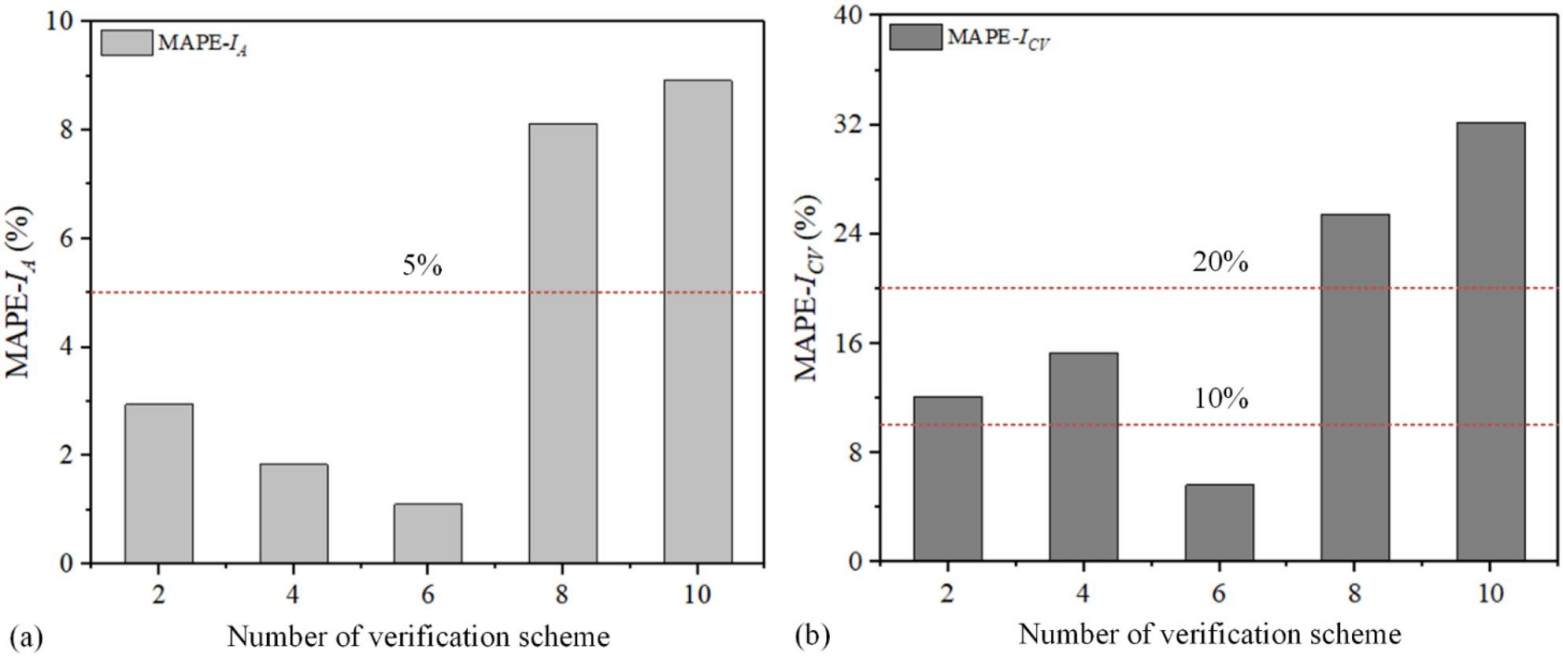
The bar chart of *MAPE*: (**a**) *MAPE-I*_*A*_, (**b**) *MAPE-I*_*A*_.

Considering the unavoidable measurement error leading to *I*_*A*_ out of tolerance, the predicted schemes {2, 4, 6, 8, 10} were verified by TUVAR experiments. And Eq. (9) is used to calculate the mean absolute percentage error (*MAPE*) of *I*_*A*_ and *I*_*CV*_. The results of verification experiments and the *MAPE* analysis of* I*_*A*_ and *I*_*CV*_ are listed in Table 5. Furthermore, the bar charts of *I*_*A*_ and *I*_*CV*_* MAPE* are displayed in Fig. 9, which could be a more intuitive comparison of *MAPE* values. Comparing with the predicted *I*_*A*_ (*I*_*A-p*_) and experimental measurement *I*_*A*_ (*I*_*A-m*_), it could be seen that the values of *I*_*A*_* MAPE* are lower than 10%. Therefore, the *I*_*A-p*_ and *I*_*A-m*_ have good consistency. In addition, the minimum *I*_*A*_* MAPE* of 1.099% is achieved, when the *I*_*A*_ is 0.018. However, the values of *I*_*CV*_* MAPE* are larger than 10%, except the Scheme 6, the values of *I*_*CV*_* MAPE* for Scheme 2 and Scheme 4 are less than 20%, and the values of *I*_*CV*_* MAPE* for Scheme 8 and Scheme 10 are larger than 20%. Therefore, the consistency of *I*_*CV-m*_ and *I*_*CV-p*_ is poor, but the minimum *I*_*CV*_* MAPE* is 5.630% when the *I*_*A*_ is 0.018. To sum up, when the *I*_*A*_ is 0.018, both the *MAPE*(*I*_*A*_) and *MAPE*(*I*_*CV*_) are minimum.

10 $$MAPE = \frac{1}{N}\sum\limits_{i = 1}^{N} {\left| {\frac{{\hat{y}_{i} - y_{i} }}{{y_{i} }}} \right| \times 100\% }$$

where $\hat{y}_{i}$ represents the predicted value of *I*_*A*_ by interpolation algorithm, *y*_*i*_ is the measured value of *I*_*A*_ by experiments.

**Table 5:** The results of verification experiments and the MAPE analysis of* I*_*A*_ and *I*_*CV*_.

Scheme	(*D*_1_, *D*_2_, *D*_3_)	(*I*_1_, *I*_2_, *I*_3_)	*I*_*A-m*_	*I*_*A-p*_	*MAPE* (*I*_*A*_)	*I*_*CV-m*_	*I*_*CV-p*_	*MAPE* (*I*_*CV*_) (%)
2	(4.140, 4.130, 4.137)	(0.0147, 0.0123, 0.0139)	0.0136	0.0140	2.941%	0.0753	0.0844	12.085
4	(4.153, 4.143, 4.143)	(0.0179, 0.0155, 0.0155)	0.0163	0.0160	1.841%	0.0711	0.0602	15.331
6	(4.160, 4.150, 4.153)	(0.0196, 0.0172, 0.0180)	0.0182	0.0180	1.099%	0.0563	0.0507	5.630
8	(4.167, 4.153, 4.147)	(0.0212, 0.0180, 0.0163)	0.0185	0.0200	8.108%	0.110	0.0820	25.455
10	(4.177, 4.160, 4.150)	(0.0237, 0.0196, 0.0172)	0.0202	0.0220	8.911%	0.135	0.0916	32.148

### Mechanical property analysis

**Figure 10:**
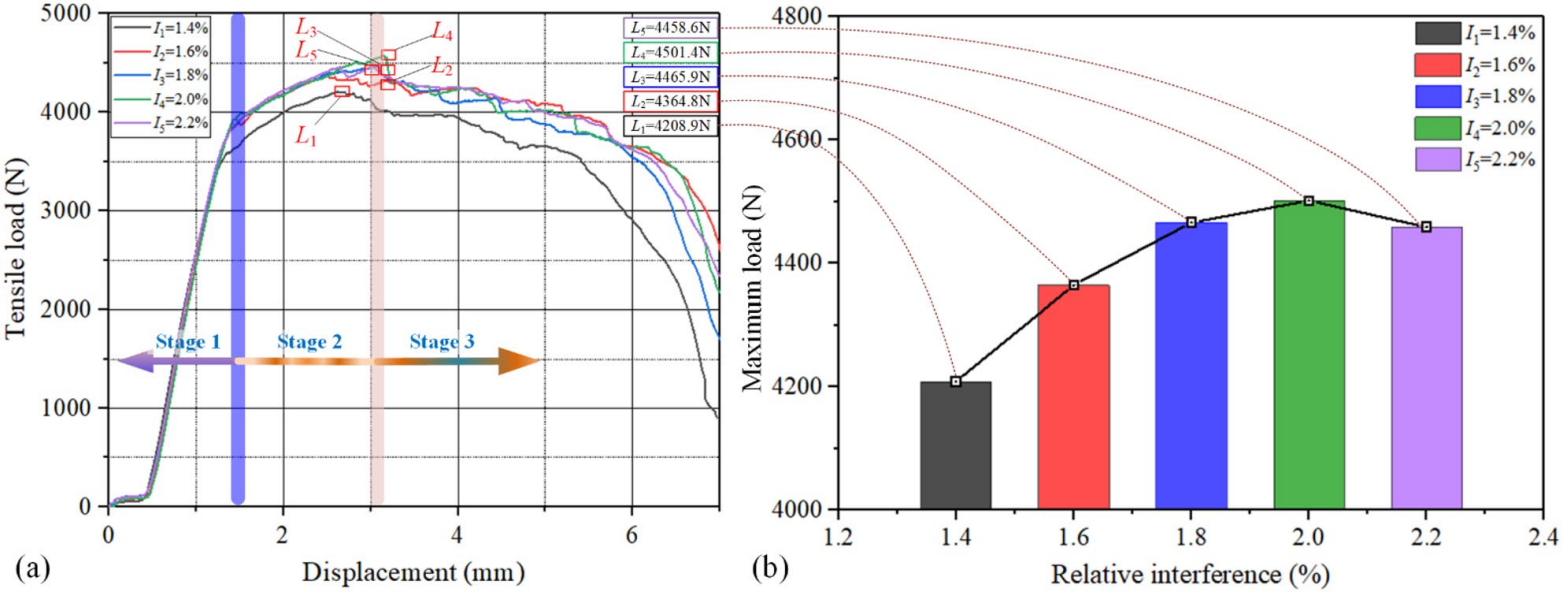
Analysis of tensile experiments: (**a**) typical average tensile curve with different *I*_*A*_, (**b**) maximum load analysis.

Tensile tests of TUVAR riveted lap joint specimens were carried out with the relative interference size *I*_*A*_ = {1.4%, 1.6%, 1.8%, 2.0%, and 2.2%}. The repeated results of tensile tests for different relative interference-fit sizes are listed in Table 6. The typical average load–displacement curves of five groups of specimens are displayed in Fig. 10a. The load–displacement curves are divided into three-stage, i.e. stage 1: hole elastic deformation, stage 2: hole plastic deformation, and stage 3: hole failure. In stage 1, the maximum tensile strength of the interference-fit sizes *I*_*A*_ = {1.6%, 1.8%, 2.0%, 2.2%} have a good consistency, and the elastic maximum tensile load is about 3800N. However, the maximum bearing load of the interference-fit size *I*_*A*_ = 1.4% in stage 1 is lower than the interference-fit sizes *I*_*A*_ = {1.6%, 1.8%, 2.0%, 2.2%}, and the elastic maximum tensile load is about 3600 N. In stage 2, it could be obtained that the tensile load of *I* = 1.4% is still less than others. The ultimate strength of different interference-fit sizes is listed and marked with red rectangular regions.

**Table 6:** The repeated results of tensile tests for different interference sizes.

Interference size (%)	Repeat 1 max load (N)	Repeat 2 max load (N)	Average max load (N)	Failure mode
1.4	4135.746	4282.106	4208.9	Pull off failure mode CFRP: resin matrix peel off, carbon fiber fracture, delamination Rivet: distortion of the mechanical head, elongation of riveted bar
1.6	4377.906	4351.729	4364.8	
1.8	4431.842	4499.971	4465.9	
2.0	4440.236	4562.564	4501.4	
2.2	4438.798	4478.462	4458.6

To straightly analyze the variation tendency of tensile strength with the different interference-fit sizes, the relationship between the relative interference-fit sizes and maximum load is displayed in Fig. 10b. It could be seen that the tensile strength increases at first and then declines with the increase of interference-fit size. There is a maximum ultimate strength when the interference-fit size *I*_*A*_ = 2.0%, and the difference in tensile strength is not significant with *I*_*A*_ = 1.8%. An appropriate interference-fit size is beneficial to the stiffness improvement of the CFRP riveted lap joints. However, the maximum tensile strength of the specimen with the interference-fit size *I*_*A*_ = 2.2% is lower than *I*_*A*_ = {1.8%, 2.0%}. This phenomenon may be attributed to the severe damage to the hole wall surface by the rivet rod expansion after the TUVAR, especially the micro-scale carbon fiber damage in local regions. Moreover, earlier researches^13, 26^ showed that micro-scale damage near the hole surface area varies with interference-fit size. As a consequence, appropriate interference-fit size can well realize a close fit between the deformed rivet shaft and the hole wall. Meanwhile, uniform interference-fit size can increase the actual contact and provide a reaction force of the hole wall, and decline the stress value and uniform stress distribution. However, the large interference-fit size (e.g. *I*_*A*_ = 2.2%) will induce delamination, fiber fracture et al., which resulted in the reduction of riveted lap joints. As a result, proper interference-fit size is beneficial to the improvement of the ultimate tensile strength of CFRP laminates riveted lap joints, whereas an excessive interference fit can reduce the maximum tensile strength.

## Conclusion

In this paper, the TUVAR experiments were carried out to investigate the effect of process parameters on the interference-fit size of the CFRP riveted lap joints. Multiple arithmetics are combined to optimize the TUVAR process parameters and quantized the interference-fit size. Meanwhile, the tensile tests were carried out to identify the accuracy of the optimized results. the main conclusions were drawn as follows:

1. Compared with conventional riveting, the P_2_ position interference-fit size of TUVAR increases, significantly, and the P_1_ position interference-fit size declines a lot. the coefficient of variance of relative interference size decreases about 9 times, and the uniformity of interference-fit size under the TUVAR improves significantly
2. The results of the quantitative weight coefficient show that process parameters have a significant effect on {*I*_1_, *I*_2_, *I*_3_, *I*_*A*_}. In addition, the vibration amplitude is the maximum weight coefficient, then the time and roughness, and the average weight coefficients are 0.497, 0.336, and 0.167, respectively
3. The *MAPE* results of the *I*_*A*_ and *I*_*CV*_ by the inverse solving algorithm and the experiment showed that the *MAPE* of the *I*_*A*_ is lower than 10%, which has a good prediction accuracy. But the *MAPE* of the *I*_*CV*_ is larger than 20% when the interference-fit size is over 2.0%, which showed that uniform interference-fit sizes of TUVAR are difficult to achieve by combining with the *MAPE* of the *I*_*A*_.
4. The results of tensile tests showed that the best interference-fit size of the riveted specimen under the TUVAR is 1.8% or 2.0%, and the maximum tensile load arrives at 4562.564 N. Compared with the interference size of the riveted specimen under conventional riveting, the tensile load improves by 6.94%.

## Data Availability

All data generated or analyzed during this study are included in this published article.
