# Author Correction: Experimental investigation and quantitative prediction in interference-fit size of CFRP riveted joints under a transversal ultrasonic vibration-assisted riveting

**DOI:** 10.1038/s41598-024-57165-0

**Published:** 2024-03-19

**Authors:** Xingxing Wang, Yunyang Shi, Haicheng Pan, Yegao Chen

**Affiliations:** 1 College of Mechanical and Electrical Engineering, Suqian University, Suqian No. 399, Sucheng District, Huanghe Street, Suqian, 223800 China; 2 College of Mechanical and Electrical Engineering, Nanjing University of Aeronautics and Astronautics, Nanjing, 210016 China

Correction to: *Scientific Reports* 10.1038/s41598-023-41578-4, published online 02 September 2023

The original version of this Article omitted an affiliation for author Xingxing Wang. The correct affiliations are listed below.

College of Mechanical and Electrical Engineering, Suqian University, Suqian No. 399, Sucheng District, Huanghe Street, Suqian, 223800, China.

College of Mechanical and Electrical Engineering, Nanjing University of Aeronautics and Astronautics, Nanjing 210016, China.

The original Article has been corrected.

